# Testosterone Therapy for the Treatment of Unexplained Anemia in Men With Hypogonadism

**DOI:** 10.7759/cureus.66887

**Published:** 2024-08-14

**Authors:** Kabeer Ali, Jay Talati, Christopher Mikulas, Austin Quan, Pramod Reddy

**Affiliations:** 1 Internal Medicine, University of Florida College of Medicine – Jacksonville, Jacksonville, USA; 2 Internal Medicine, University of Florida College of Medicine, Gainesville, USA

**Keywords:** testosterone replacement, unexplained anemia, andropause, low testosterone, male hypogonadotropic hypogonadism

## Abstract

Decreased testosterone levels are often under-recognized as a cause of anemia in males with hypogonadism. Men, as a subset, are less likely to seek medical care, especially those who struggle with complex psychiatric and social conditions, where they may lack full autonomy. Increasing testosterone levels leads to erythrocytosis by elevating erythropoietin and soluble transferrin receptor levels and suppressing hepcidin and ferritin levels. While practice guidelines on testosterone therapy for hypogonadism exist, there are no large-scale, randomized clinical trials assessing the use of testosterone replacement therapy in men with hypogonadism to evaluate its effect on anemia. Testosterone replacement therapy is also not wholly benign, and patients may be at increased risk for nonfatal cardiac arrhythmias, venous thromboembolism, and acute kidney injury. We explore two cases of patients with similar prior medical history, both of whom were found to have hypogonadism and anemia that were not otherwise explained. Both patients experienced significant improvement in their anemia following testosterone supplementation.

## Introduction

Globally, anemia presents a significant health burden and is estimated to have a prevalence between seven and 24 percent of the general population [1]. Anemia can lead to symptoms of lightheadedness, fatigue, dyspnea, loss of consciousness, and increased hospitalization. Causes of anemia include iron deficiency, chronic inflammation and disease, renal insufficiency, alcoholism, myelodysplastic syndrome, and vitamin B-12 and vitamin B-6 deficiency. Nearly a third of adults above the age of 65 are unable to identify the cause of their diagnosed anemia [2]. One potential cause to consider in cases of unidentified anemia is testosterone deficiency. Testosterone deficiency may be attributed to normocytic anemia, and nearly 15% of older men with hypogonadism experience anemia [3].

Secondary analyses of a substudy of a testosterone trial reported that testosterone supplementation may correct normocytic anemia in patients with hypogonadism and reduce the incidence of anemia in men with hypogonadism who are not anemic [3]. Testosterone replacement has been clinically demonstrated to have similar hemoglobin-increasing effects as erythropoietin-stimulating agents among patients with hypogonadism [3]. It is postulated that this increase in hemoglobin occurs due to increased erythropoietin transcription, reduced hepcidin transcription, and improved survival of erythrocytes [4]. However, there have been no large-scale randomized clinical trials (RCTs) that study the relationship between testosterone replacement therapy in men with hypogonadism and its impact on anemia as a primary outcome. Here, we present a case series of patients who presented to the hospital with hypogonadism and had improved anemia following initiation of testosterone replacement therapy.

## Case presentation

Case 1

**Figure 1:**
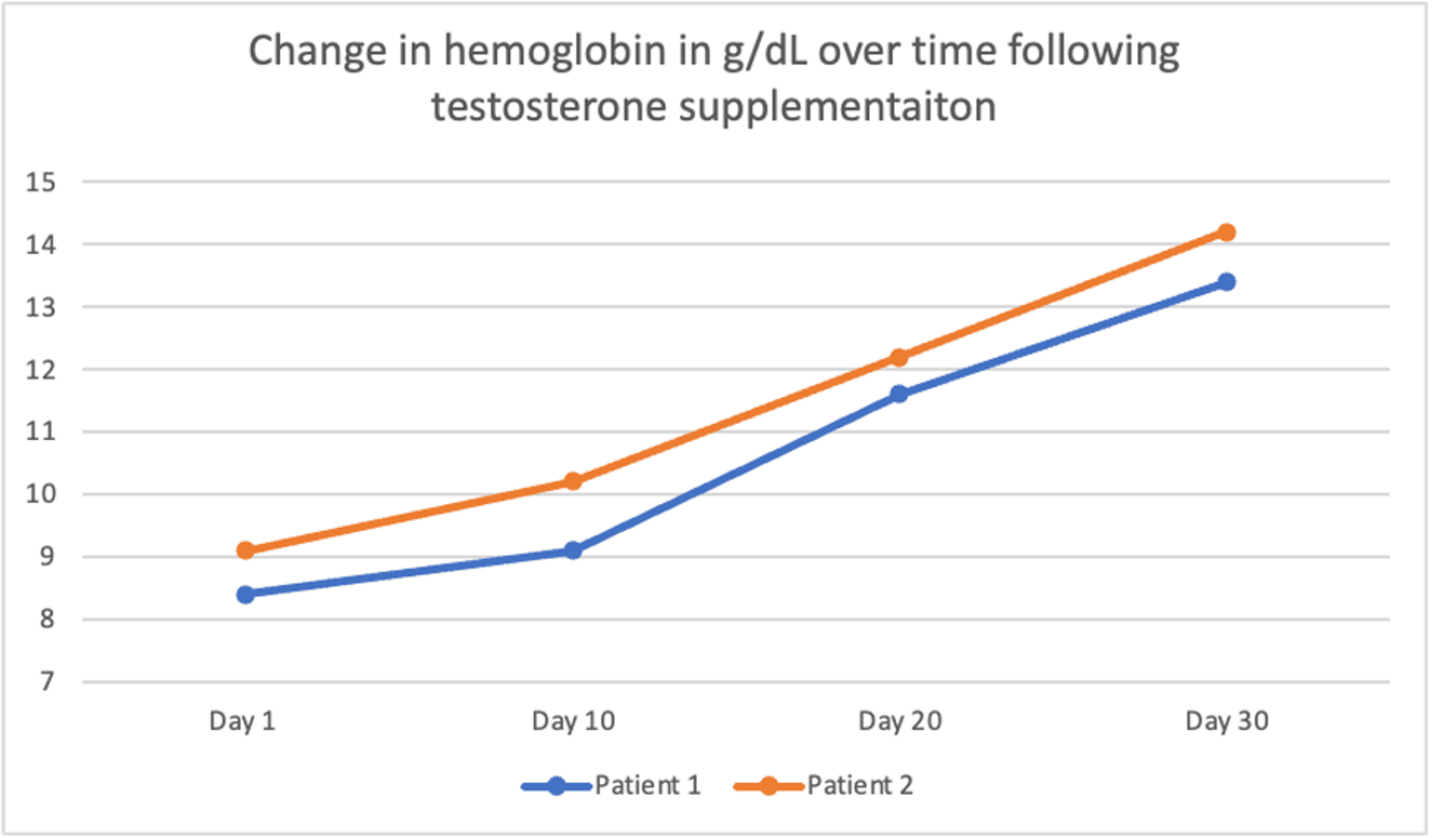
Graphical representation of the patients’ trend in hemoglobin levels following testosterone supplementation.

A 58-year-old male with a past medical history of schizophrenia, traumatic brain injury, hypertension, and chronic obstructive pulmonary disease (COPD) presented to the emergency department after suffering a mechanical ground-level fall and was admitted for inpatient psychiatric evaluation. The patient lived in a behavioral health facility and reported being adherent to his medications, which include levothyroxine, atorvastatin, allopurinol, lisinopril, and risperidone. He did not have a history of anemia before this hospital admission, with his previous laboratory investigations from one and a half years prior showing a hemoglobin level consistently greater than 13 grams per deciliter (g/dL). Physical examination was remarkable for pale mucus membranes and testicular volume of approximately 10-12 milliliters using a Prader orchidometer. Laboratory investigations are highlighted in Table 1, which revealed normocytic anemia with hemoglobin of 8.4 g/dL and mean corpuscular volume (MCV) of 91 femtoliters (fL). Folate level was 17.9 nanograms per milliliter (ng/ml), and vitamin B-12 level was 601 ng/ml, both within normal limits. His iron panel showed normal serum ferritin and iron saturation but decreased transferrin and total iron-binding capacity (TIBC), which is consistent with anemia of chronic disease. The patient’s renal and liver function tests were within normal limits. Given the patient’s hypogonadism, it is possible the patient’s normocytic anemia was secondary to hypogonadism. Free and total testosterone were decreased at five picograms per deciliter (pg/dL) and 215.7 nanograms per deciliter (ng/dL), respectively. Previous free testosterone was measured ten years ago and was 42 g/dL, within the normal range. The patient was started on a 200 mg intramuscular testosterone cypionate to be taken every 14 days. Repeat hemoglobin measured one month after beginning testosterone therapy was markedly improved at 13.4 g/dL with MCV 94 fl (Figure 1), with the patient also reporting symptomatic improvement in shortness of breath.

**Table 1:** Comparison of the patient’s laboratory investigations, including anemia workup before and after testosterone therapy, with reference ranges included. MCV: mean corpuscular volume.

Characteristics	Patient 1	Patient 2	Reference range
Medical history	Schizophrenia, traumatic brain injury, hypertension, hypothyroidism, gout, chronic obstructive pulmonary disease	Schizophrenia, hypertension, hypothyroidism, seizure disorder, dyslipidemia, coronary artery disease	
Hemoglobin before testosterone therapy	8.4 g/dL	9.1 g/dL	14.0-18.0 g/dL
MCV	91 fL	93 fL	82-101 fL
Ferritin	320 ng/mL	164 ng/mL	30.0-400.0 ng/mL
Iron saturation	19%	18%	20-55%
Total iron-binding capacity	225 mcg/dL	269 mcg/dL	261-390 mcg/dL
Transferrin	177 mg/dL	212 mg/dL	200-360 mg/dL
Folate	17.9 ng/ml	19.3 ng/mL	4.4-19.9 ng/mL
Vitamin B-12	601 ng/mL	462 ng/mL	243-894 ng/mL
Serum free testosterone	5 pg/dL	2.6 pg/dL	6.6-18.1 pg/dL
Serum total testosterone	215 ng/dL	179.3 ng/dL	264.0-916.0 ng/dL
Dose of testosterone given	200 mg intramuscular every 14 days	200 mg intramuscular every 14 days	
Hemoglobin one month after testosterone therapy	13.4 g/dL	14.2 g/dL	14.0-18.0 g/dL
MCV one month after testosterone therapy	94 fL	96 fL	82.0-101.0 fL

Case 2

A 60-year-old male with a past medical history of schizophrenia, hypertension, hypothyroidism, seizure disorder, dyslipidemia, and coronary artery disease was admitted to the hospital for decompensated schizophrenia after being found in the street with disorganized speech. His physical examination revealed multiple abrasions, pallor, and a testicular volume of 10-12 milliliters using a Prader orchidometer. His outpatient medications were levothyroxine, atorvastatin, and haloperidol. However, he was not adherent to these medications. His workup for an infectious, toxic, and metabolic cause of his symptoms was found to be negative. His laboratory investigations were notable for a similar picture (Table 1) with hemoglobin of 9.1 g/dL and MCV of 93 fL. A previous hemoglobin measurement from four years prior showed a hemoglobin of 13.2 g/dL. His iron panel was inconclusive but more suggestive of anemia of chronic disease with a normal ferritin of 164 ng/mL, total iron-binding capacity of 269 micrograms per deciliter (mcg/dL), transferrin 212 milligrams per deciliter (mg/dL), and iron saturation of 18 percent. His vitamin B-12 level was 462 ng/mL, and folate was 19.3 ng/mL, within normal limits. A similar approach was used, with 200 mg testosterone cypionate given intramuscularly, with repeat dosing every two weeks. After one month, his repeat complete blood count showed a rise in hemoglobin to 14.2 g/dL with MCV 93fL (Figure 1). The patient was stabilized, and haloperidol was switched to the long-acting injectable paliperidone, with the aim of better psychiatric control leading to better long-term follow-up.

## Discussion

Andropause, also known as androgen decline in the aging male (ADAM), typically begins as early as 40 years. This is characterized by a gradual decline in testosterone levels, which continues progressively with age. Testosterone deficiency may manifest clinically with symptoms of decreased libido, loss of body hair/beard growth, hypogonadism, erectile dysfunction, fatigue, and poor concentration [5]. It occurs as a consequence of reduced testicular production of testosterone in the setting of advancing age [6]. Both patients in this case were approximately 20 years post-andropause, and it is not surprising that there was testosterone deficiency. On the other hand, it is interesting that both patients had similar medical co-morbidities, specifically a diagnosis of schizophrenia, hypothyroidism, and hypertension. Both patients had poor control over their psychiatric conditions. There is a multifaceted link between schizophrenia and hypogonadism due to intrinsic hypothalamic-pituitary-gonadal (HPG) axis dysfunction and also from neuroleptic side effects.

Many patients with psychiatric conditions exhibit hyperprolactinemia and gonadal dysfunction [7]. The majority of antipsychotics prescribed to these patients, such as haloperidol and risperidone, increase prolactin levels by acting as antagonists at dopamine (D2) receptors. Prolactin inhibits gonadotropin-releasing hormone (GnRH), leading to reduced testosterone levels. This is evident in studies showing high rates of hypogonadism in patients on antipsychotic treatment [8]. During puberty, hemoglobin increases in boys, mirroring changes in testosterone concentrations [9]. Boys with delayed puberty have been demonstrated to have hemoglobin levels similar to girls and prepubertal boys [9]. Testosterone treatment of boys who have delayed puberty has been shown to normalize hemoglobin levels [9]. This provides support that a decline in testosterone with aging may negatively affect erythropoiesis. A longitudinal study in Italy provides further evidence by demonstrating that older men and women with testosterone levels in the lowest quartile were more likely to develop anemia than those subjects in the other three quartiles over a follow-up period of three years [10]. In the case above, we have a stable patient who incidentally had normocytic anemia found after a mechanical ground-level fall. The iron panel, thyroid, and B-12 labs were found to be within normal limits, so the unexplained asymptomatic anemia was postulated to occur from testosterone deficiency as the patient was found to have hypogonadism on physical exam. The use of a testosterone patch improved hemoglobin back to normal levels.

Hemoglobin increase secondary to elevated testosterone levels is proposed to be mediated by various pathophysiologic mechanisms, including elevated erythropoietin and soluble transferrin receptor levels associated with suppressed hepcidin and ferritin levels [11]. Additionally, testosterone has been associated with increased erythroblast number and survival [12]. Clinical trials with men receiving exogenous testosterone demonstrate increased erythropoietin levels for the first three months following treatment, followed by a return to baseline erythropoietin levels at six months despite increased hemoglobin levels from prior, suggesting that testosterone increases the erythropoietin “set point” to a higher hemoglobin level [13]. The patient was continued on a testosterone patch for the prevention of recurrent normocytic anemia. Treatment with supplemental testosterone may put patients at increased risk of nonfatal cardiac arrhythmias and acute kidney injury, so it is essential to monitor renal and cardiac function regularly with primary care physicians. However, studies have not shown an association with testosterone and major adverse cardiovascular events [3].

Although there are no randomized clinical trials (RCTs) that analyze the direct effect of testosterone supplementation in treating anemia, there is evidence for the potential therapeutic benefits of testosterone replacement therapy. Studies in mice models have shown that testosterone supplementation normalized older mice’s iron status and stimulated splenic erythropoietic activity [14]. There have been some studies demonstrating the association of testosterone replacement therapy with adverse cardiovascular events in men with hypogonadism [3]. The TRAVERSE study had 5,204 participants across the United States who met the criteria of being between the ages of 45-80, having low testosterone concentration, hypogonadism, and cardiovascular disease [3]. Secondary analysis of this trial revealed that 815 of these men had anemia. There was a significant increase in the proportion of men who had anemia correction with testosterone replacement therapy compared to placebo [3]. Even in patients without anemia, a significantly lower proportion of men developed anemia in the future with testosterone replacement therapy compared to placebo use [3].

Similarly, another observational study evaluated 58 patients who received 1,000 mg of testosterone undecanoate. There was a significant decrease in anemia prevalence and a considerable increase in erythropoietin levels after 18 weeks [12]. Another notable study is a 2020 randomized control trial of 95 hypogonadal men, showing a statistically significant increase in hemoglobin in men with unexplained anemia [15]. These studies, combined with our case series, provide further evidence for the implementation of testosterone supplementation in elderly patients with hypogonadism and decreased testosterone levels to treat and prevent normocytic anemia. Furthermore, we encourage providers to regularly monitor hemoglobin levels upon initiating testosterone supplementation and consider measuring serum testosterone levels in older men with unexplained anemia. However, a large-scale longitudinal RCT is needed to provide further insight into the efficacy of testosterone replacement therapy in treating unexplained normocytic anemia in elderly patients compared to a placebo.

## Conclusions

Andropause, which can begin as early as age 40, has had more attention in recent years as more attention has been given to men’s health. A significant portion of men are still reluctant to seek healthcare, especially for issues surrounding genitourinary and mental health, until medical conditions are advanced. Hypogonadism remains an under-recognized cause of anemia in older males and should be part of a comprehensive anemia workup. Further research evaluating the effects of testosterone replacement in hypogonadal patients with anemia as a primary outcome is needed to assess the safety and efficacy of such treatments.
